# Three-Dimensional Multidetector CT for Anatomic Evaluation of Orbital Tumors

**DOI:** 10.1155/2013/674230

**Published:** 2013-10-31

**Authors:** J. Matthew Debnam, Rory R. Mayer, Bita Esmaeli, Jeffrey S. Weinberg, Franco DeMonte, Nandita Guha-Thakurta

**Affiliations:** ^1^Department of Radiology, Section of Neuroradiology, The University of Texas MD Anderson Cancer Center, 1400 Pressler Street, Unit 1482, Houston, TX 77030, USA; ^2^Department of Neurosurgery, Baylor College of Medicine, 1709 Dryden Rd., Suite 750, Houston, TX 77030, USA; ^3^Orbital Oncology and Oculofacial Plastic Surgery Program, Department of Plastic Surgery, The University of Texas MD Anderson Cancer Center, 1515 Holcombe Blvd, Unit 1488, Houston, TX 77030, USA; ^4^Department of Neurosurgery, The University of Texas MD Anderson Cancer Center, 1515 Holcombe Blvd, Unit 0442, Houston, TX 77030, USA

## Abstract

Intricate resection and complex reconstructive procedures often required for primary and metastatic orbital tumors are facilitated by accurate imaging. A three-dimensional (3D) image can be reconstructed from source axial multidetector computed tomography (MDCT) images to visualize orbital tumors. To assess the utility of 3D images in this setting, the 3D images were reconstructed retrospectively for 20 patients with an orbital tumor and compared to two-dimensional (2D) orthogonal MDCT studies. Both types of images were assessed for their capacity to show the bony orbital walls and foramina, extraocular muscles, and optic nerve in the orbit contralateral to the tumor and, in the affected orbit, the extent of the tumor and its relationship to normal orbital contents and associated bone destruction. 3D imaging is most informative when axial images are acquired at 1.25 mm collimation. The optic nerve, extraocular muscles, and well-circumscribed orbital tumors were well visualized on 3D images. On 3D imaging, tumor-associated destruction of the lateral and superior orbital walls was fairly well demonstrated and that of the inferior and medial walls was not. The 3D images provide the surgeon with a comprehensive view of well-circumscribed orbital tumors and its relationship to extraocular muscles, exiting foramina, and the superior and lateral walls.

## 1. Introduction

Surgical intervention for primary and metastatic tumors of the orbit and its vicinity, including the periorbital soft tissue and sinonasal cavity, requires intricate resection and complex reconstructive procedures [1–8]. Routine preoperative radiographic evaluation for orbital tumors typically involves magnetic resonance imaging (MRI) and computed tomography (CT). Several authors have concluded that high-quality preoperative imaging studies are essential in preoperative planning [5–7, 9]. Advanced imaging techniques related to multidetector computed tomography (MDCT) allow generation of three-dimensional (3D) data sets [10–12]. Prior studies have demonstrated the usefulness of 3D MDCT imaging for evaluation of facial fractures [13], detection of intraocular and orbital foreign bodies [14], and assessment of facial asymmetry [15]. Others have described the technique in preparation for craniofacial surgery for facial deformities [16, 17], facial fractures [18], and nasal anomalies [19].

In planning for surgical resection and reconstruction of orbital tumors, it is imperative that the imaging provides accurate delineation of the orbital lesion and its extent, relationship to orbital structures, and associated bone destruction. As the 3D imaging technique for the evaluation of normal orbital anatomy and orbital tumors and the relation of tumors to orbital structures has not, to the best of our knowledge, been described, we sought to identify the technical imaging parameters that maximize effectiveness and determine how 3D imaging could best be utilized for preoperative imaging of the orbit and orbital tumors. We, therefore, compared the capacities of 3D imaging and two-dimensional (2D) MDCT for the preoperative evaluation of orbital tumors by assessing their visualization of orbital walls, extraocular muscles, and orbital foramina and their relation to the orbital tumor. The purpose is not to suggest that 3D imaging replaces 2D MDCT or MRI of the orbit, rather to determine if 3D imaging is technically feasible and under what circumstance would this technique be utilized.

## 2. Methods

### 2.1. Patient and Clinical Data

The Institutional Review Board of The University of Texas MD Anderson Cancer Center approved this retrospective study and waived the requirement for informed consent. Consecutive MDCT studies of the orbit in patients with primary or metastatic disease within the orbit or in the periorbital or sinonasal region were reviewed for this study. The imaging studies and clinical data were reviewed by two neuroradiologists (JMD and NGT) in a consensus fashion.

Three-dimensional images were reconstructed from axial MDCT data obtained from patients with orbital tumors. To evaluate the clinical utility of 3D images, we first assessed the appearance of the unaffected side, including the orbital walls, the exit foramina, and the extraocular muscles and optic nerve. We then evaluated the affected orbit, including visualization of the tumor, its relation to the adjacent extraocular muscles and optic nerve, and underlying bone destruction. 

### 2.2. MDCT Technique (Orthogonal)

The MDCT parameters were as follows: 1.25 to 5 mm slice thickness, field of view 180–250, kVp 120–140, and mA 180–220. Intravenous contrast medium was administered in 19 of 20 cases. Fifteen studies were acquired at 1.25 mm thickness, three studies at 2.5 mm thickness, and two studies at 5 mm thickness. Imaging was performed in the axial plane; the images were transferred to a GE Advantage workstation (GE Medical Systems, Milwaukee, WI) and reformatted by a trained technologist in the orthogonal planes, that is, coronal and sagittal.

### 2.3. 3D Reformatting

The axial data were transferred to an Aquarius iNtuition workstation (Version 4.4.6; TeraRecon, Foster City, CA) for postprocessing. Two-dimensional imaging in three-orthogonal planes and reformatted 3D images were available for viewing and editing. The 3D image was cropped to remove the intracranial compartment and periorbital soft tissues deemed uninvolved by the interpreting radiologist (JMD). The 3D images could be viewed in both soft tissue and bone windows settings; the soft tissues were first evaluated and then subtracted to allow visualization of the underlying bony structures. The rating system used to compare 2D orthogonal MDCT and 3D images is described in the following sections.

### 2.4. Characterization of the Contralateral Orbit

The superior, medial, inferior, and lateral walls of the orbit on the side opposite the tumor were evaluated on both 2D and 3D images to determine how well they were visualized. Each image was rated from 0 to 3 indicating how much of the orbital wall was adequately visualized (3, ≥75%; 2, 50–75%; 1, 25–50%; 0, ≤25%). Each image also was rated on whether the superior orbital fissure, the optic canal, the extraocular muscles (including the superior, medial, inferior, and lateral rectus muscles), and the optic nerve of the orbit on the contralateral side could be visualized in their entirety (0 = not visualized, 1 = visualized). 

### 2.5. Evaluation of 3D Images

In evaluating the utility of the 3D images, the 2D orthogonal images were used as the reference standard to which the 3D images were compared. Each 3D image was rated as to whether the full extent of the tumor could be visualized and whether it fully demonstrated the relationship of the tumor to the optic nerve and to the extraocular muscles (0 = not visualized, 1 = visualized). For lesions with underlying destruction of the orbital wall, the bone windows of the 3D images, with the soft tissues subtracted out, were rated on whether the bone destruction could be visualized (0 = not visualized, 1 = visualized).

## 3. Results

### 3.1. Patient and Clinical Data

We selected 20 consecutive patients for inclusion in this retrospective study (11 men and 9 women; age range 17–80 years, mean 47.1 years, median 46.5 years) with a component of the tumor involving the orbit. The epicenter of the tumor was located in the orbit (*n* = 15), periorbital region (*n* = 3), or sinonasal cavity (*n* = 2). The 20 tumors comprised adenoid cystic carcinoma (*n* = 11), neuroendocrine carcinoma (*n* = 3), squamous cell carcinoma (*n* = 2), salivary duct carcinoma ex-pleomorphic adenoma (*n* = 1), inverted papilloma (*n* = 1), synovial cell sarcoma (*n* = 1), and orbital pseudotumor/idiopathic orbital inflammation (*n* = 1). Three-dimensional images were available for all 20 cases. Biopsy results were available for 19 of the 20 patients; the exception was a patient (no. 20) with presumed orbital pseudotumor, which responded to steroids. Demographic and clinical data for the 20 patients, including treatment, are provided in Table 1.

### 3.2. Characterization of the Contralateral Orbit

**Figure 1:**
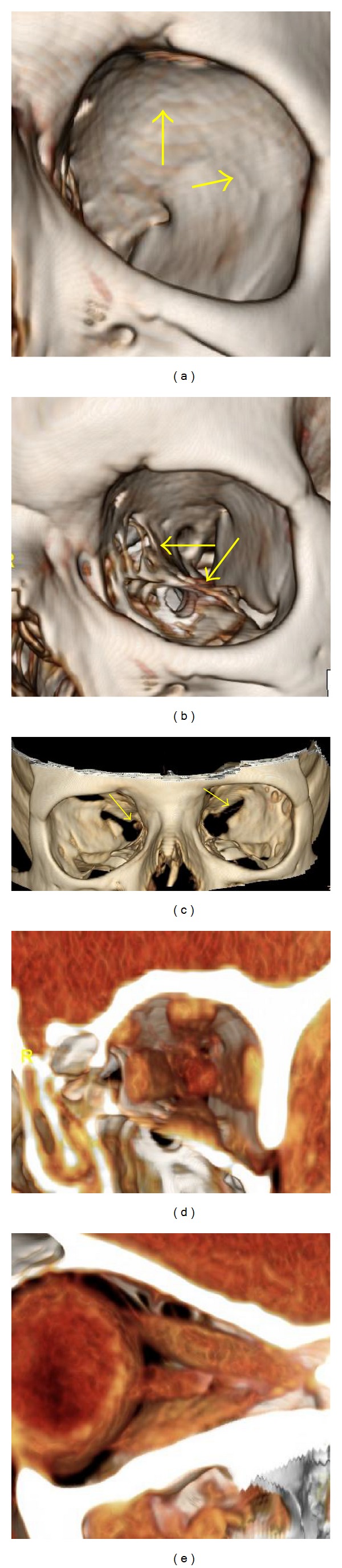
Normal anatomy of the bony orbit, globe, extraocular muscles, and optic nerve. (a) 3D image, bone window: smooth contour of the superior and lateral orbital walls (arrows). (b) 3D image, bone window: “spongiform” appearance of the lamina papyracea and orbital floor (arrows). (c) 3D image, bone window: demonstration of the optic canal and superior orbital fissure (arrows). ((d), (e)) 3D image, normal appearance of the extraocular muscles and optic nerve.

On the 2D orthogonal MDCT images, the orbital walls on the contralateral side received the following average ratings: 1.9 (superior wall), 2.9 (lateral wall), 0 (medial wall), and 0.1 (inferior wall) (Figure 1). On the 3D images, the mean ratings were 1.6, 2.9, 0, and 0.1, respectively. All images were rated 1 for visualization of both the superior orbital fissure and the optic canal.

In all 20 cases, the 2D MDCT images were scored 1 for visualization of the optic nerve and extraocular muscles. On 3D images, 15 orbits received a score of 1, each acquired at 1.25 mm collimation. Five cases received a score of 0, all acquired at 2.5 or 5 mm collimation.

### 3.3. Visualization of Tumor Extent, Relationship to Adjacent Soft Tissue Structures, and Bone Destruction on 3D Images

**Figure 2:**
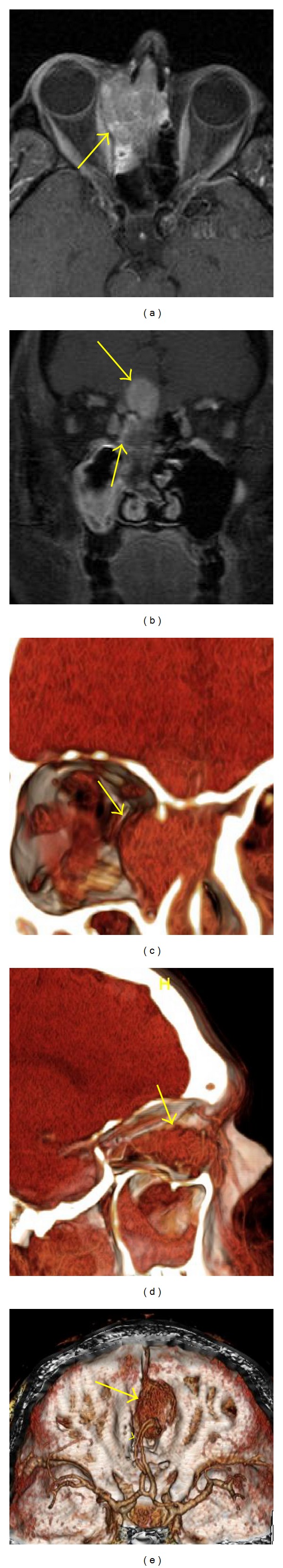
46-year-old man with sinonasal neuroendocrine carcinoma. (a) Axial T1 postcontrast MRI shows the tumor in the right sinonasal cavity with extension through the lamina papyracea into the left orbit (arrow). (b) Coronal T1 postcontrast MRI shows tumor extending into the anterior cranial fossa (arrow). (c) 3D image gives a frontal view of the sinonasal tumor with orbital extension and medial displacement of the medial rectus muscle (arrow). (d) 3D image that has been rotated; the lateral orbital wall, optic nerve, and extraocular muscles have been removed to demonstrate a lateral view of the tumor (arrow). (e) 3D image giving a view from above the tumor in the anterior cranial fossa (arrow). Note the anterior cerebral arteries (arrowhead).

For visualization of tumor extent (Figure 2), 12 images received a score of 1 and the remaining eight were scored 0. Five of the eight cases that scored 0 were acquired at 5 mm or 2.5 mm collimation. Two cases (patients 14 and 20) were scored 0 because they involved perineural spread of tumor along the second and third divisions of the trigeminal nerve, so that complete extent of tumor was not well demonstrated. The final cases were performed without intravenous contrast (patient no. 3).

Fourteen of the 20 cases received a rating of 1 for visualization of the relationship of the tumor to the orbital contents on the 3D image. The other six cases received a rating of 0. Two of these six cases were acquired at 5.0 mm and one at 2.5 mm collimation. The other three cases were the two that involved perineural spread and the one that was without contrast such that the relationship with orbital contents was not well demonstrated.

**Figure 3:**
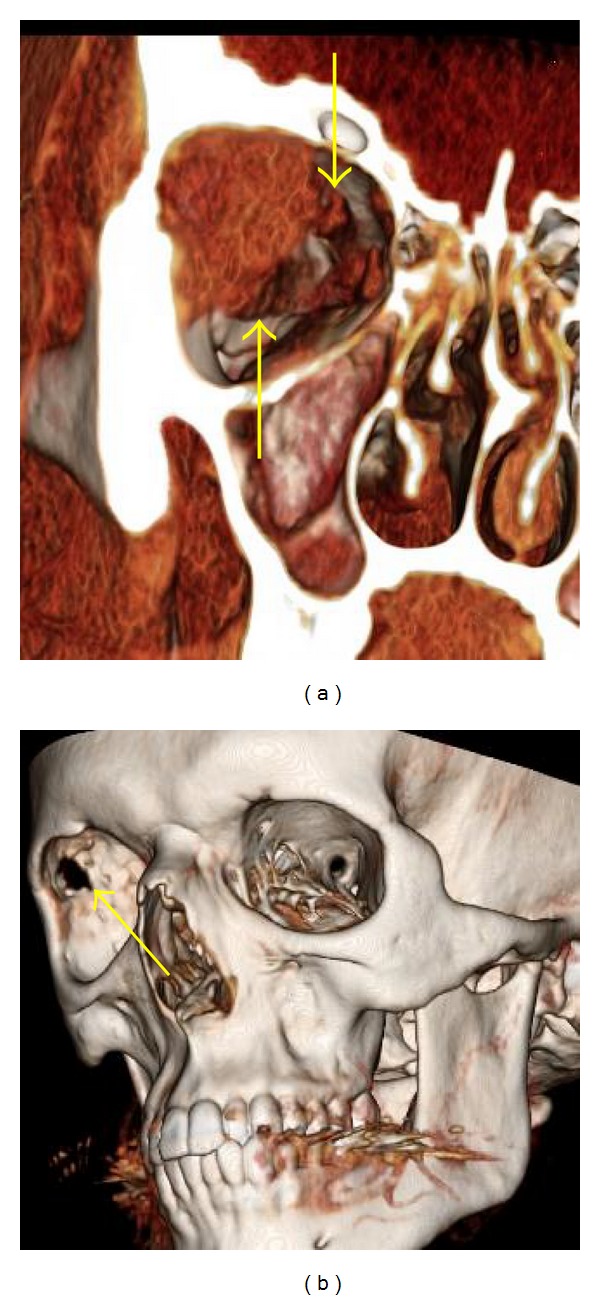
Involvement of adjacent bony structures by tumor demonstrated by 3D imaging. A 17-year-old man with poorly differentiated lacrimal gland adenocarcinoma “salivary duct carcinoma ex-pleomorphic adenoma.” (a) 3D image, soft tissue window: soft tissue tumor and relation to the extraocular muscles (arrows). (b) 3D image, bone window (soft tissue subtracted). The tumor has been removed to reveal the underlying bone destruction (arrow).

**Figure 4:**
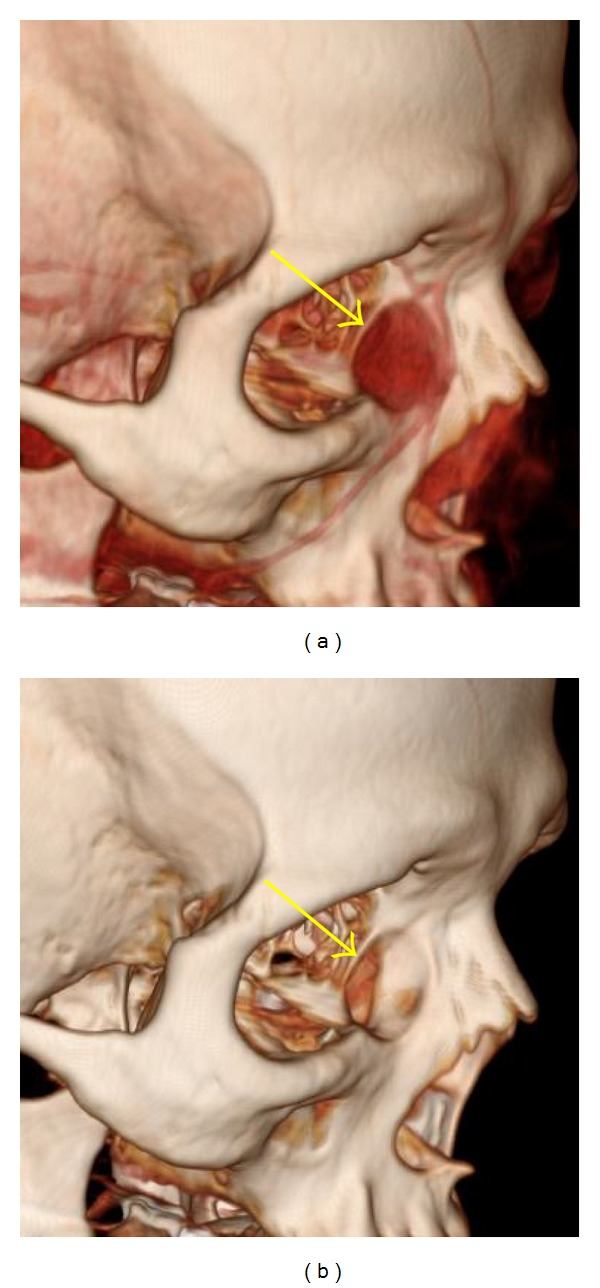
Involvement of adjacent bony structures by tumor demonstrated by 3D imaging. A 53-year-old woman with lacrimal sac inverted papilloma. (a) 3D image, soft tissue window: tumor in the left lacrimal sac (arrow). (b) 3D image, bone tissue window. The tumor has been removed to show underlying bone remodeling (arrow).

Tumor-associated bone destruction (rating of 1) was present on imaging 18 cases (Figures 3 and 4). On 2D MDCT, this was well demonstrated in 16 cases; however, the extent of the bone destruction was not well visualized in two cases, both acquired at 5 mm collimation. On the 3D images, bone destruction was well visualized in 11 of 18 cases and not visualized in the remaining seven cases. Two of these seven cases were those acquired at 5 mm, and in two more, both acquired at 2.5 mm collimation. The other three cases involved the medial wall of the orbit. Tumor involved the superior orbital fissure and optic canal in four cases, each of which received a score of 1 on the 2D MDCT images. Only two of these lesions were demonstrated with 3D images, both acquired at 1.25 mm collimation; the other two were not well visualized, and both were acquired at 2.5 mm collimation.

## 4. Discussion

Our findings suggest that 3D images of the orbit generated from 2D MDCT allowed visualization of orbital contents and orbital tumors comparable to 2D orthogonal images when acquired at 1.25 mm collimation following the administration of intravenous contrast. Visualization of orbital contents at 2.5 mm and 5.0 mm collimation and without contrast was inadequate. Of significant clinical importance is that this technique is best applied to well-circumscribed tumors, including those studied as well as other circumscribed lesions such as meningiomas and nerve sheath tumors. The technique is inadequate in the demonstration of perineural tumor extension. Further imaging with fat-suppressed MR with and without contrast is necessary in these patients. The 3D images offered fairly adequate visualization of the bony contours of the lateral orbital wall but less adequate visualization of the orbital roof. Small areas of thinning of the orbital roof and lateral walls were visualized as normal findings; these should not be mistaken for areas of bone destruction. The surgeon and radiologist should evaluate the proximity of the orbital tumor to a suspected defect in the orbital roof, assess for spread of tumor to the intracranial compartment through the orbital roof, and be aware that these apparent defects may not be related to bony thinning rather than destruction. The superior orbital fissure and optic canal were well visualized with the 3D images at any collimation.

Cockerham et al. [6] illustrated the importance of good-quality preoperative imaging in selecting the surgical approach to orbital tumors. Lesions of the anterior orbit are usually treated with a transorbital approach, while lesions in the deep orbital apex are most often approached through a craniotomy. Three-dimensional imaging provides the surgeon with a comprehensive view of the tumor and orbital contents and may benefit surgical planning, when interpreted in addition to standard 2D MDCT and MRI. In contradistinction to the standard orthogonal views with 2D MDCT, which are static, 3D images may be cropped to remove irrelevant anatomy and rotated so that the surgeon may assess orbital tumors in any plane deemed necessary, including the direct surgical approach to the tumor, improving preoperative surgical planning. We are not suggesting that the 3D images replace standard 2D MDCT but rather that in certain circumstances 3D images may be beneficial in the preoperative evaluation of orbital tumors.

One limitation of the 3D images that we identified is their inability to reliably assess associated bony destruction along the orbital floor and medial wall. Because of the thinness of the lamina papyracea and the orbital floor relative to the roof and lateral walls, these take on a “spongiform” appearance on 3D images with multiple apparent defects. Therefore, caution must be exercised while evaluating for bony destruction of the lamina papyracea and orbital floor, and standard 2D orthogonal images may be required for evaluation of these regions. However, we did not find the 2D orthogonal images to be better than 3D images due to the relative thin nature of the lamina papyracea and floor, especially with thicker section imaging. A potential concern of the 3D technique is the radiation exposure to the lens in the eye; however, lens doses during “high-resolution” CT is below the threshold dose that causes a cataract [20]. The 3D images are reformatted by computer manipulation without the need for additional imaging, and therefore do not increase radiation exposure.

Tumors such as adenoid cystic carcinoma of the lacrimal gland may involve important intraorbital structures such as the extraocular muscles and optic nerve, destroy the bony walls of the orbit, and extend through the orbital foramina or into the intracranial compartment [21, 22]. Orbital exenteration may improve locoregional control for adenoid cystic carcinoma of the lacrimal gland [23, 24]; however, several studies have demonstrated that orbital exenteration may not alter survival outcomes [2, 25]. Three-dimensional images such as those assessed in this study could be used in future studies to correlate the degree of bony destruction with intraoperative findings and perhaps help select cases for whom a globe-sparing surgical approach may be possible. 3D images may also prove beneficial in planning of adjuvant radiation therapy for patients with adenoid cystic carcinoma of lacrimal gland in whom an orbital exenteration is not performed.

In this study, we found that 1.25 mm collimation was superior to imaging at thicker collimation. In the future, larger prospective studies are needed to determine the preoperative benefit of this technique to orbital surgeons and neurosurgeons. This will require a determination of the accuracy of 3D images in defining the location and size of the tumor and the amount of actual bone destruction present when comparing 3D images to MR imaging and clinical and operative findings. It will also be necessary to compare the findings on 3D imaging with more conventional 2D CT, as well as MR imaging in determining the surgical approach and whether any modifications were made on the basis of the 3D images. Another interesting utility for 3D CT imaging is in the teaching of orbital anatomy to surgical residents and fellows. 

## 5. Conclusion

MDCT images obtained at 1.25 mm collimation are best suited for 3D reconstruction. The 3D images provide the surgeon with a comprehensive view of a well-circumscribed orbital tumor and may be utilized to demonstrate the relationship to adjacent soft tissue structure, exiting foramina, and the superior and lateral walls. Assessment of the inferior and medial walls and the presence of perineural extension of tumor are incomplete.

## Figures and Tables

**Table 1:** Baseline patient demographic and clinical data.

Patient number	Age/Sex	CT collimation (mm)	Tumor pathology	Tumor location	Treatment
1	43/M	2.5 mm	ACC	Lacrimal gland	Exenteration
2	43/F	2.5 mm	ACC	Lacrimal gland	Exenteration
3*	47/F	1.25 mm	ACC	Lacrimal gland	Exenteration
4	43/F	5.0 mm	ACC	Lacrimal gland	Exenteration
5	69/F	1.25 mm	ACC	Lacrimal gland	Exenteration
6	24/F	1.25 mm	ACC	Lacrimal gland	Resection
7	29/M	1.25 mm	ACC	Lacrimal gland	Exenteration
8	36/M	1.25 mm	ACC	Lacrimal gland	Exenteration
9	17/M	1.25 mm	Carcinoma ex-pleomorphic adenoma	Lacrimal gland	Exenteration
10	25/F	1.25 mm	Pleomorphic adenoma	Lacrimal gland	Resection
11	57/M	1.25 mm	ACC	Lacrimal gland/sac	Exenteration
12	53/F	1.25 mm	Inverted papilloma	Lacrimal sac	Resection
13	51/M	1.25 mm	Synovial sarcoma	Intraconal	Exenteration
14	56/M	1.25 mm	Invasive basal cell carcinoma	Periorbital/infraorbital nerve	Chemoradiation
15	80/F	1.25 mm	Basaloid squamous carcinoma	Medial canthus	Exenteration
16	67/F	5 mm	Invasive squamous carcinoma	Medial canthus	Exenteration
17	59/M	2.5 mm	Neuroendocrine carcinoma	Intraconal	Exenteration
18	51/M	1.25 mm	Invasive squamous carcinoma	Medial orbit/sinonasal	Resection
19	46/M	1.25 mm	Neuroendocrine carcinoma	Medial orbit/sinonasal	Chemoradiation
20	46/M	1.25 mm	Pseudotumor**	Orbital apex	Steroids

ACC: adenoid cystic carcinoma; NA: not applicable.

*No intravenous contrast.

**Responded to treatment with steroids.
